# Using an experience based design approach to advance health literacy in Ireland

**DOI:** 10.1016/j.dialog.2026.100340

**Published:** 2026-08-11

**Authors:** Hannah Goss, Maeve Murray, Talent Nyamakope, Mairead Carney, Craig Smith, Sarah Meegan, Sarahjane Belton, Stephen Behan

**Affiliations:** a School of Health and Human Performance, Dublin City University, Dublin, Ireland; b Insight Centre for Data Analytics, Dublin City University, Dublin, Ireland; c Sláintecare Healthy Communities, Ireland; d Community, Social Development & Research, Dublin City Council, Dublin, Ireland; e Community and Integrated Development, Mayo County Council, Mayo, Ireland; f Department of Public Health & Epidemiology, School of Population Health, Royal College of Surgeons in Ireland, Dublin, Ireland

**Keywords:** Health education, Health behaviour, Community, Co-design, Disadvantage

## Abstract

Health literacy supports people, communities, and organisations in addressing noncommunicable diseases and their determinants, taking meaningful action to reduce risk factors, and support health and wellbeing for all. Addressing the issue of low health literacy has the potential to increase health, health equity and health system effectiveness by building citizens' capacities for health. The Sláintecare Healthy Communities programme was launched in 2021 in Ireland focussing specifically on promoting health and wellbeing at the community level, and improving health literacy is a key area of focus for this programme. This study aimed to work with stakeholders, in two Sláintecare Healthy Community areas, through a four-stage reflective process to discover, define, develop, and deliver innovative recommendations to improve health literacy within their communities. Two co-design workshops, adopting an adapted double diamond design approach (DDDA) took place in Spring 2024, one in each case study area. Sixty-eight stakeholders engaged in two DDDA workshops, undertaking tasks in same stakeholder and mixed stakeholder groups. A series of recommendations were co-created, with stakeholders voting for their preferred recommendations to refine this list in each area. These recommendations highlighted the need for effective early intervention, improved health infrastructure, and engaging with the community to connect, support, and sustain existing health literacy related initiatives. This is a novel example of health literacy stakeholder engagement in Ireland, and critically, it provides a tangible example of the process and outcomes of a collaboration between stakeholders and researchers which can be applied beyond these contexts.

## Introduction

Enhancing health literacy is crucial, not only for individual wellbeing, but also for bolstering public health and ensuring the long-term sustainability of healthcare systems. Health literacy is concerned with the capacities of people to meet the complex demands of health in a modern society [1], and a critical, modifiable determinant of health [2]. Health literacy supports people, communities, and organisations in addressing non-communicable diseases and their determinants, taking meaningful action to reduce risk factors, and support health and wellbeing for all [3]. Addressing the issue of low health literacy has the potential to increase health, health equity and health system effectiveness through building citizens' capacities for health [4], [5]. In line with this, in 2022, the WHO published a report entitled ‘Health literacy development for the prevention and control of noncommunicable diseases’ which aimed to support countries and partners build health literacy responsive environments and interventions across countries, sectors and stakeholders [6]. The critical role of organisations, societal structures, and resources to support health literacy at a systems level has become increasingly recognised in modern discourse [3], [7].

In Ireland, low health literacy levels have been reported in adults [8], [9] suggesting that more needs to be done to support health literacy promotion in the region. Globally, a social gradient in health literacy and associated consequences for health has been observed [4], [10]. Analysis of Irish data from the European Health Literacy Survey (HLS-EU) suggests strengthening health literacy within the population through targeted means can be considered as a mechanism for addressing socio-economic disparities in health and healthy behaviours [9]. Ireland is recognised as one of the only countries in the European Union without universal healthcare coverage for all citizens, offering a ‘two-tiered system’ which contributes to, and exacerbates, rising health inequalities [11]. While there are organisations and policies in place aiming to reduce the health inequality gradient, the responsibility for health, and subsequently health literacy, often falls to and between multiple stakeholders.

The Sláintecare Healthy Communities Programme, which was launched in 2021 by Sláintecare Healthy Ireland in the Department of Health, working with the Health Service Executive (HSE) and local authorities and community agencies, focuses specifically on promoting health and wellbeing at the community level. This involves initiatives aimed at preventive healthcare, health promotion, and addressing the social determinants of health. The programme takes a place-based approach to tackling health inequalities with a focus on the determinants of health and has identified 19 communities of focus across Ireland following an evidenced based process to identify local areas in which health and wellbeing risk factors are particularly concentrated. In these areas, local authorities have employed Local Development Officers and have been provided funding to improve public realm and fund locally identified projects to improve health and wellbeing. The Department of Health are working across Government with the Sláintecare Oversight Group to coordinate interventions and policy responses. This involves initiatives aimed at preventive healthcare, health promotion, and addressing the social determinants of health. With this in mind, as a modifiable determinant of health, health literacy is a key area of focus for the Sláintecare Healthy Communities Programme. At this stage of the Sláintecare Healthy Communities Programme, developing evidence-informed pilot projects, focused on a smaller number of identified communities, is a strategic priority.

One of these projects was a health literacy needs analysis, focussing on one urban Sláintecare Healthy Community (Finglas and Cabra) and one rural Sláintecare Healthy community (Mayo) in Ireland [12]. Driven by local health literacy needs, strengths and challenges identified by Murray et al. (in submission; in submission), this study proposed to co-design a series of recommendations with stakeholders, suggesting targeted actions for the future. Among other advantages, co-produced research can: i) recognise, value, and utilise experiential knowledge, ii) support the prioritisation of research topics, aims, and questions by people who are typically excluded from or marginalised in the research process, iii) address inequities in power and amplify marginalised or excluded voices through the recruitment of a diverse range of research participants and iv) deliver impactful research that can provide solutions to problems and positively influence people's lives [13]. Therefore utilising a co-design process to check, challenge, collaborate and create recommendations for health literacy solutions is an invaluable process to “disrupt the status quo” (p.20) and build the case for developing health literacy system capacity leveraging user engagement [3].

A significant scientific gap remains regarding how place-based, community-level structural barriers interact with individual health literacy capabilities within highly centralised or unequal healthcare systems like Ireland's. While traditional frameworks often default to educational or individual-focused solutions, contemporary health literacy models demand a transition towards health literacy-responsive systems [3]. This manuscript addresses this specific gap by offering empirical insights into how regional stakeholders conceptualise system-level adaptations. It bridges the divide between theoretical framework assumptions and localised, actionable realities, contributing a replicable, experience-based co-design blueprint for public health policy.

A specific method of experience-based co-design is the Double Diamond Design Approach (DDDA) that has been used to develop service improvements in health and social care [14], [15]. The DDDA is a framework often used in design thinking to tackle and solve complex problems. Co-design, within the context of the Double Diamond, refers to involving end-users, stakeholders, and relevant parties throughout the design process, ensuring that their perspectives, needs, and insights are integrated into the solution. With DDDA, stakeholders progress through a four-stage reflective process to discover, define, develop, and deliver an innovative solution to a problem. Therefore, the aim of this study was to revise the health literacy strengths, barriers, needs, and generate recommendations for health literacy supports in each Sláintecare Healthy Community of focus, using an adapted DDDA.

## Methods

### Participants

Purposive sampling was used to identify participants for these workshops. As part of a wider project, recruitment was initiated through Sláintecare Healthy Community Local development officers, working with communities in rural (Mayo) and urban (Finglas and Cabra) areas to recruit participants with lived experience of the health literacy strengths, needs and issues encountered in the Sláintecare Healthy Communities focus areas.

The stakeholders were specifically invited to involve a representation of the different demographics, local authorities and a range of experiences, in each respective case study area, this included participants who had previously been involved in earlier phases of the wider project and had indicated a willingness to continue their participation [16]. Stakeholders were contacted via telephone and/or e-mail and invited to register online for the workshop in their respective area. To actively mitigate inherent power asymmetries between institutional service providers (e.g., HSE medical staff, local authority executives) and service users (e.g., community members, marginalised groups), precise structural safeguards were embedded directly into the workshop architecture. Phase One utilized homogeneous groupings (same-stakeholder tables) so that participants could safely debate and define their lived experiences without risk of being overridden by institutional authority figures. Each table was overseen by a neutral academic researcher trained in participatory research methods. Facilitators utilized a semi-structured guide containing explicit prompts to distribute airtime equitably, disrupt dominant narratives, and amplify quieter voices. The ultimate prioritisation step used physical dot voting (dotmocracy), decoupling the merit of an initiative from the institutional or social status of its advocate. Despite these mitigations, certain systemic limitations to participation persisted. Due to localised transport deficits in rural regions and technology requirements for online pre-registration, highly isolated individuals, transient populations, and those experiencing extreme socioeconomic exclusion were underrepresented, with community agency advocates stepping in to voice their perspectives. All participants gave informed consent to take part in the study. Ethical approval was granted by Dublin City University (DCUREC/2023/156), and this study was carried out in accordance with this approval, and in line with the Standards for Reporting Qualitative Research (see supplementary material).

### Overview

Two co-design workshops took place in March 2024, one in each case study area. At the start of the day, participants were provided with written information on the project to date (this included two options: a one-page summary, and/or a more detailed five-page overview). A brief presentation was delivered at the start of the day, which provided a description of the different work packages undertaken of the wider project which highlighted some key considerations. The presentation finished outlining the aims of the co-design workshop process and the format of day.

The DDDA was selected over other participatory methodologies (e.g., traditional focus groups or Delphi panels) because its structural alternating phases of divergent and convergent thinking are uniquely optimised for resolving complex, multi-stakeholder healthcare issues. It ensures that systemic root causes are explored extensively before narrowing down to practical solutions. In this study, the original British Design Council model was adapted to accommodate a time-constrained single-day format, this included retaining to core components of Discover (diverging to map challenges), Define (converging on core priority issues), and Develop (diverging to brainstorm solutions), and Deliver (converging to select final actions).

**Fig. 1:**
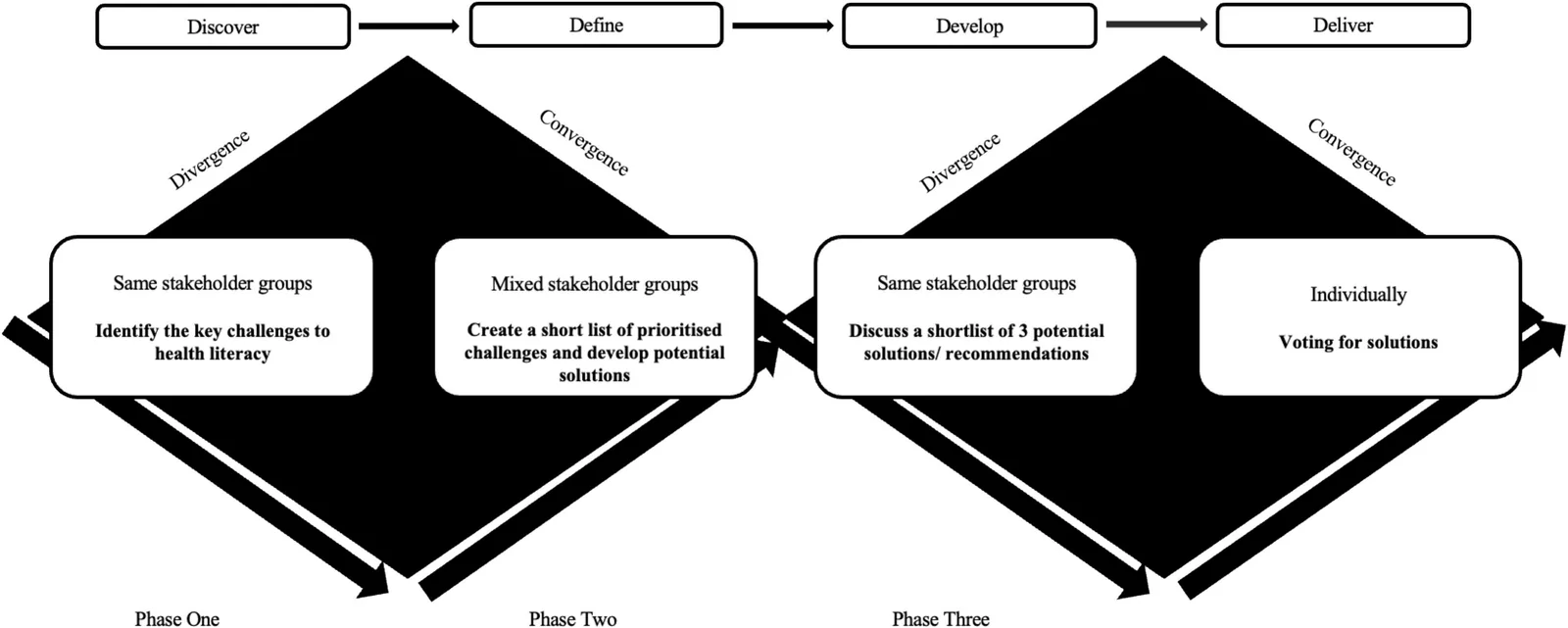
Overview of the Adapted DDDA used within each workshop.

An adapted DDDA was divided into three phases with one or more tasks per phase (outline; Fig. 1). Within each phase, stakeholders worked in the same stakeholder groups (e.g. physical activity promotion) or mixed stakeholder groups. All discussions were recorded via Dictaphones on each table. Stakeholders were allocated to mixed stakeholder groups, ensuring at least one member from each of the initial same stakeholder groupings were present in each group (See Table 1). Dependent on attendance numbers, in some cases more than one member of the initial same stakeholder group may have been present in the mixed stakeholder group. In Mayo, owing to travel disruption, the decision was made to combine two similar, same stakeholder groups to maximise discussion, resulting in five same stakeholder groups. Each group had a researcher (experienced with qualitative and/or participatory research) present on their table to facilitate discussion (using a semi-structured interview guide), point towards provided resources outlining previous work, and to take notes. Flip chart paper, pens, post-it notes were provided on each table for the participants to engage with as they wished.

**Table 1:** Participant characteristics described within mixed stakeholder groupings.

Mayo	Stakeholder Group	Description	Number of Participants
Group 1 *n* = 8	Service Provider	Community Development	2
	Service User	Language and Culture	2
	Service User	Community Service	3
	Service User	Community Member	1
Group 2 *n* = 6	Service Provider	Family Services	1
	Service Provider	HSE/ Health Services	1
	Service Provider	Sport, Health and Wellbeing Services	1
	Service User	COPD Chronic Illness Community Member	2
	Service User	Community Member	1
Group 3 *n* = 5	Service Provider	Sport, Health and Wellbeing Services	1
	Service Provider	Healthy Ireland	1
	Service Provider	Mental Health Services	2
	Service User	Community Member	1
Group 4 *n* = 4	Service Provider	Community Development	2
	Service User	Community Member	2
Group 5 *n* = 7	Service Provider	Community Development	2
	Service Provider	Public Participation Network	1
	Service Provider	Transport Development	1
	Service User	Community Older Person's Council	2
	Service User	Community Member	1
**Finglas and Cabra**	**Stakeholder Group**	**Description**	**Number of Participants**
Group 1 *n =* 9	Service Provider	HSE/ Health Services	9
Group 2 *n =* 9	Service Provider	Education Services	3
	Service User	Education Services	6
Group 3 *n* = 5	Service Provider	Community Development Services	4
	Service User	Community Older Person's Council	1
Group 4 *n* = 6	Service Provider	Drug and Alcohol Services	2
	Service Provider	Traveller Support Services	1
	Service Provider	Children Services	1
	Service User	Drug and Alcohol Services	1
	Service User	Community Development Services	1
Group 5 *n* = 5	Service User	Community Member	4
	Miscellaneous	n/a	1
Group 6 *n* = 4	Service Provider	Sport, Health and Wellbeing Services	2
	Service User	Community Member	2

In Phase One, within the same stakeholder groups, the focus was to refine the key challenges to health literacy in their community. Reflecting on challenges identified previously [16], [17] participants were encouraged to reflect on the clarity and breadth of these challenges, while starting to prioritise the importance of these challenges within their local communities. Within phase one, participants were actively encouraged to avoid considering potential solutions. Moving to Phase Two, participants were asked to physically move to mixed stakeholder groups. This phase encompassed two tasks: 1) Identifying five main challenges to health literacy development in their communities and 2) Developing suggestions to support health literacy development in their communities. Participants were encouraged to consider their discussions in Phase One, the resources required to deliver on their health literacy recommendations, who this would impact, and what outcomes these recommendations could achieve. In Phase Three, participants returned to their initial same stakeholder groupings and were tasked with creating a shortlist of three potential recommendations. Phase Three condensed the Develop and Deliver components using structured table exercises, physical movement between mixed stakeholder groupings, and a rapid democratic voting technique (dotmocracy) to establish immediate community priority consensus within the session. At the end of this Phase, the researcher summarised the recommendations decided on by their same stakeholder groups, and participants were invited to vote for the recommendations they felt best achieved the aim of the brief, using a dotmocracy approach. Each participant received three votes (dot stickers) with a maximum of two votes being allowed for any one recommendation. After voting, the lead researcher concluded the day, reminded participants of how this process was fitting in with the wider project, and informed them that they would all be sent a copy of the lay summary of the project's final report.

## Results and discussion

This is a novel example of health literacy stakeholder engagement in Ireland. Critically, it provides a tangible example of the process and outcomes of a collaboration between stakeholders, researchers and the Sláintecare Healthy Communities Programme. The findings of this study highlight the importance of working in partnership with communities in developing shared solutions. Globally, there are a growing number of tangible examples that detail different approaches to meaningfully engage with community members [18]. Within the current study, the adapted DDDA enabled community members from different backgrounds to share, discuss and debate a common goal; improving health literacy. Critically, the process was developed mindful of the power dynamics between different community members and the research team and aimed to recognise the expertise and lived experience of all. Some examples of this included skill recognition, feedback opportunities, and shared decision making throughout the workshop(s).

Within this study, following final voting by stakeholders on the presented group solutions at phase three, a number recommendations were generated for each co-design workshop. These co-design recommendations are presented here as they were presented during the workshop sessions, with some additional information presented in brackets for comprehensiveness. Audio-recordings were not transcribed due to poor sound quality, but participant notes, feedback, and facilitator notes and reflections were used to provide this additional information where needed. Because poor acoustic quality limited full verbatim audio transcription, a transparent, multi-source framework was engineered to ensure rigorous qualitative analysis and traceability. Raw data consisted of participant-written flip charts, post-it notes, individual feedback forms, and facilitator summary logs completed immediately post-workshop. Three members of the research team (MM, HG, SM) conducted a collaborative qualitative content analysis. Items were organised electronically, systematically cross-referenced against table notes, and categorised chronologically matching the workshop phases. Member-checking was executed at the end of Phase Three by verbally presenting synthesised recommendations back to tables for validation before voting. Discordant interpretations between researchers were resolved via collaborative consensus reviews, maintaining a clear trail from raw artifact to final recommendation. The final recommendations from each workshop have been displayed in a table format in order of participant preference voting. It should be noted that where similar recommendations were made by different groups within each area, these recommendations were synthesised after the workshop by the research team, and votes for these ideas were collated. To demonstrate transparency regarding participant prioritisation, the finalised co-designed recommendations are presented below in order of voting preference, with explicit percentage proportions of the total votes cast included. Table 2 illustrates the Finglas and Cabra Sláintecare area recommendations and added descriptions. Table 3 illustrates the Mayo Sláintecare area recommendations and added descriptions. A defining conceptual insight from these co-design workshops is that when disadvantaged or underserved communities are tasked with generating solutions for health literacy, they intuitively build strategies targeting structural determinants of health and system responsiveness, rather than individual cognitive capabilities. Stakeholders explicitly argued that an individual's health literacy is context-dependent, directly related to the complexity of the environment they are forced to navigate. For example, if clinical staff turnover is unsustainably high, or geographic distances to clinics are insurmountable due to non-existent public transport, traditional educational interventions were obsolete. Therefore, expanding basic access, stabilising local medical staffing, and smoothing institutional communication pathways are not external to health literacy, they are the fundamental pre-requisites of a health-literate system.

**Table 2:** Finglas and Cabra Sláintecare healthy community recommendations.

Recommendation	Percentage of total votes	Additional description provided by groups
Community Engagement	35%	Health related community events Community kitchen/ garden/ supermarket/ health eating/ cooking/ growing are of specific interest Schools' engagement- signposting to social and mental health benefits Peer to peer learning
Infrastructure (long term)	18%	Funding supports- critical to help people get funding Primary care access Schools and early interventions need to be long term
Early Intervention	13%	Such as a paid school post to cover all aspects of health literacy e.g. Physical Education, mental health, hygiene etc. Social maintenance scheme- using existing and new local clubs/ health centres/ community groups
Resource Development (both digital and physical)	11%	Easy to understand Tailored to different populations
Community Hub	10%	Classes to train locals to deliver training (community champions)

**Table 3:** Mayo Sláintecare healthy community recommendations.

Recommendation	Percentage of total votes	Additional description provided by groups
Community Outreach Officer	22%	[Who should lead this] Local authorities? HSE? [Should be a…] Salaried position
Funding Networks	19%	Coordinating the disconnect (of funding schemes) Central person (known in the community) to help with funding Prioritise sustainable grant schemes
Preventative – access where people are at	18%	Mobile primary care unit– focusing on women's health, people's general health/ population health Health check evenings that are sustainable, linked back to the mobile unit etc. Can be advertised using Mid-West radio to share information
Local Healthcare Professionals	15%	Community healthcare nurses needed locally Promote use of district hospitals more (take pressure of GPs and district nurses) Stepdown beds needed Develop primary healthcare centres appropriately Promote appropriate use of pharmacies (take pressure off GPs)
Community Activities	9%	Social and physical health should both be considered Intergenerational approaches Communication is key consideration

Notably, there were similarities and differences in the proposed recommendations across each community setting, with these spanning across what would be considered functional, interactive and critical health literacy [19], and advocating for individual to policy level change [3]. As suggested by Sørenson et al. [3] investing in health literacy system capacity can lead to a more sustainable and scalable effort, moving beyond solely depending on changing organizational or individual behaviour. This reflects a shift in wider health literacy research and practice [2], [3], [20]. Within both areas, it was established that any initiatives to improve health literacy would require engagement with community members and should take advantage and align with existing established programmes. Specific local initiatives that implicitly and indirectly linked to health literacy were highlighted throughout the workshops. This included, for example, adult literacy programmes and physical activity promotion clubs (where the activity was deemed far less relevant than the who and how this club was facilitated). It is widely recognised that effective health literacy programmes empower communities, particularly in underserved or vulnerable areas, by equipping individuals with the knowledge and skills needed to improve their health and wellbeing [6], [9]. Despite this, within the workshops, advertising, timing, transport, and sustainability of such programmes were mentioned as potential barriers to their long-term effectiveness. As has been acknowledged in previous European wide research, at a policy level, there is a need to support and expand existing community health literacy initiatives [5]. This will require increased investment in community-based initiatives, particularly in the communities of greatest need. Evaluation/monitoring is a crucial requirement to sustain and scale up these existing initiatives. Recent work by Chu and colleagues [20] identified and synthesised several organizational health literacy responsiveness assessment tools that could be used to support this. However, many of the tools were developed for healthcare settings, and required support from a researcher to be administered. Methods for assessing individual health literacy have also been reviewed [21], [22], but recommendations from the WHO include the suggestion to develop locally relevant strategies for surveillance, evaluation and impact assessment [6]. In Ireland, advancements have been made to co-design an assessment of health literacy for adolescents [23], but there is a need to consider assessment of health literacy in other age groups, and in other population groups of specific need. As was highlighted in this study, health literacy is context specific, making the development of valid, reliable and feasible tools a challenge.

In Finglas and Cabra, there was also an emphasis on recommendations that supported health education and early intervention spanning across school and community settings. This reiterates the importance of health literacy as a preventative approach to health promotion. Despite a shift within the academic literature to viewing health literacy from a more ‘top-down’, systems approach, within Finglas and Cabra in particular, individuals in the community still called for the need for early intervention, focussing on individual capacities and responsibilities. Aligning with the WHO action area aiming to ‘Incorporate health literacy-responsive practice into health education curricula and continuing professional development’ (p.g.37), early intervention with young people could be achieved by integrating health literacy within existing curricula [6]. The suggestions of schools as an environment to sustainably and proactively support health literacy development was explicitly mentioned, and voted for, by stakeholders in Finglas and Cabra. In Ireland, there is a clear opportunity to integrate health literacy norms, values and practices under the recently introduced ‘Wellbeing’ programme [24]. Health literacy development in young people, and specifically within schools has received increasing attention internationally [25], [26], [27], [28], [29], [30] and in Ireland specifically [31] in recent years, although there are challenges to this. In later life stages, by promoting preventive care and improving the management of ongoing health conditions, higher health literacy can decrease the reliance on emergency care services and reduce overall healthcare costs [3]. Exploring the data from Ireland collected as part of the 2011 European Health Literacy Survey, improving health literacy was suggested to have the greatest impact in those from lower social status groups, such as the identified Sláintecare Healthy Communities across Ireland, and subsequently reduce the current social gradient in the prevalence of long-term chronic health conditions, smoking, and hospital service utilisation [9]. Although this analysis was fairly recent, this is based on 2011 data and may not take into account more recent efforts to improve health literacy, or more recent events that may have exasperated health inequalities (e.g COVID-19, the cost-of-living crisis, housing conditions etc.). As a result, health literacy could be viewed as a piece of the puzzle, but it is not the only piece, and wider improvements to health care services are desperately needed.

In respect to the rural location of Mayo, improvements to healthcare access was recommended to support health literacy, with proposed solutions reflective and contextualised to a rural population. Indeed, in both communities, poor health infrastructure was a consistent topic of discussion. Individuals highlighted a clear lack of accessibility to, and availability of, a variety of health care services. This included the need for increased availability of medical appointments; an increased number of healthcare providers servicing the local community; the provision of more realistic appointment times to allow for travel; and a decrease in turnover of staff in medical settings which individuals felt resulted in ‘disjointed’ care. Improving health literacy across Ireland and beyond is not merely a healthcare concern, but a social imperative that can lead to better health outcomes, more effective utilisation of the healthcare service, and a healthier nation overall. While this is a broad and ambitious aim, health literacy is a broad concept that encompasses a wide variety of capacities that can support this endeavour [32]. To work towards achieving this aim however, building health literacy responsive health systems that understand and reflect community needs and distinct characteristics is crucial (WHO, 2022).

A further suggestion from those involved in the workshops across both areas was a call for regular community events, that are widely advertised, which would provide individuals with an opportunity to easily engage in a wide range of health related educational and social activities in a non-clinical setting. Within Mayo in particular, this was recognised as an opportunity to also reduce social isolation. Previous research has cited social support as a key practice in contributing to the health literacy of older adults as well as their communities [33]. De Wit et al. [33] categorised this social support as; emotional (e.g. sharing experiences), instrumental (e.g. tangible aid), informational (e.g. advice and information), and appraisal (e.g. information for self-evaluation). In the current study, the hope was that these health related community events could empower community members to manage and navigate their specific health needs. Previous research has found social support to improve health literacy [34], [35], so this may be a particularly relevant initiative to pursue in these communities, extending and exemplifying the shared community spirit evident in both workshops. These events were also seen as a potential way of sharing resources and training for and with the local community health ‘champions’, who many participants spoke so highly of as advocates for health literacy within their communities. The importance of these ‘champions’ was so well recognised that in Mayo, participants suggested there should be a paid, dedicated, roles (such as community outreach officers), with a responsibility for health literacy.

Ultimately, to translate these participatory co-design insights into actionable public health practice, there are several explicit structural interventions for policy makers and health service administrators to consider. At a policy level, the integration of health literacy within the national curriculum, and reforming funding architecture, (e.g. from hyper-competitive, short-term funding cycles towards rolling multi-year core operational grants) are two key suggestions. At a community level, continued funding for local outreach officers as dedicated, non-clinical system navigators, is warranted. Furthermore, in rural catchments characterised by severe public transport deficits, wide-spread and sustained funding and deployment of mobile primary healthcare clinics to carry out routine non-emergency care, localised screening, and clear signposting directly within remote towns would be of huge benefit. Many of the suggestions from this participatory work could be linked to the WHO Health Literacy recommended action areas [36], further strengthening the need to integrate, implement, and evaluate these strategies in practice to improve health literacy outcomes.

### Strengths and limitations of the study

This study, to the authors' knowledge, is the first to deploy a co-development methodology with multiple stakeholders invested in developing health literacy in Ireland. This was a flexible, reflective, and engaging process which could be adapted to a wide variety of topics. As project methods for data collection strove to be equitable and target hardly reached (as opposed to ‘hard to reach’) community members, challenges were observed in recruitment during a timebound study. Sampling of community members from both areas was established through networks of relationships with Sláintecare Healthy Communities. While these methods supported the time restrictions in completing the project, inevitably there are community members we will have not engaged with in this process. In particular, in the development of the co-design workshops limitations around lead in time and equity of access to materials, resources and a central workshop location were observed due to the geographical descriptors of each Sláintecare area. Further examples of potential barriers were the registration process of the workshops, and the informed consent process required by the research institute. In addition, due to the timeframe it was not possible to complete a typical, full DDDA [14], [15]. Meaning that the full list of recommendations were not presented back to participants after attending the workshops or in the timeframe of this pilot project, although collaboration with the communities is ongoing. These findings should not be accepted as a comprehensive, nor final, list of recommendations (but are not intended as such). Community engagement in the implementation, evaluation, and ongoing development of these recommended health literacy responsive actions should be an iterative collaborative process.

### Recommendations for future research

In line with recent calls from other researchers [37] findings in this study support the need for future longitudinal health literacy research to establish causal relationships and explore diverse geographical, socioeconomic, cultural and health system contexts. In practical terms, existing and new health literacy research and initiatives require sustainable, long-term funding to enable this. Such research will be crucial to understand how health literacy interacts as a pathway, mechanism, mediator for health outcomes in different communities. Furthermore, methodologically, future research could look to replicate this approach in different contexts to 1) develop this experience-based co-design process and 2) identify community specific, and potentially nationally and internationally, consistent and diverse recommendations for health literacy responsive actions.

## Conclusion

The findings from this study underscore the importance of community-driven recommendations for health literacy development in the Sláintecare Healthy Community areas of Finglas, Cabra, and Mayo, and beyond. The emphasis on health education and early intervention within schools and community settings in Finglas and Cabra highlights the need for sustained engagement and resource sharing among local ‘champions’. In Mayo, the focus on accessing funding and improving the evaluation of existing programs reflects the challenges faced by rural communities in supporting health literacy.

The study also reveals the necessity of building health literacy system capacity to create sustainable and scalable efforts, moving beyond individual behavioural changes. Poor health infrastructure and accessibility issues were consistently identified, reiterating the need for health literacy as a preventive approach. The integration of health literacy into educational curricula, especially through the ‘Wellbeing’ programme in Ireland, presents a significant opportunity for early intervention in young people.

Furthermore, promoting preventive care and managing chronic health conditions through improved health literacy can reduce reliance on emergency services and overall healthcare costs. The study calls for increased investment in community-based initiatives and the development of locally relevant strategies for assessing health literacy. Regular community events and social support networks are also recommended to enhance health literacy and reduce social isolation.

Overall, improving health literacy is a multifaceted endeavour that requires responsive health systems reflecting community needs. This study highlights the potential for meaningful engagement with community members to co-create solutions, thereby fostering more equitable health outcomes and empowering underserved populations.

## CRediT authorship contribution statement

**Hannah Goss:** Writing – review & editing, Writing – original draft, Supervision, Project administration, Methodology, Funding acquisition, Formal analysis, Data curation, Conceptualization. **Maeve Murray:** Writing – review & editing, Project administration, Investigation, Formal analysis, Data curation. **Talent Nyamakope:** Writing – review & editing, Project administration, Methodology, Conceptualization. **Mairead Carney:** Writing – review & editing, Project administration, Methodology, Conceptualization. **Craig Smith:** Writing – review & editing, Data curation, Conceptualization. **Sarah Meegan:** Writing – review & editing, Formal analysis, Data curation, Conceptualization. **Sarahjane Belton:** Writing – review & editing. **Stephen Behan:** Writing – review & editing, Funding acquisition, Data curation, Conceptualization.

## Clinical trial number

Not applicable.

## Funding

This work was funded by Sláintecare Healthy Communities. At the time of study completion, Talent Nyamakope and Mairead Carney were Sláintecare Healthy Communities Officers, employed by their respective local authorities.

## Declaration of competing interest

The authors declare that they have no competing interests.
